# Use of the SONET Score to Evaluate High Volume Emergency Department Overcrowding: A Prospective Derivation and Validation Study

**DOI:** 10.1155/2015/401757

**Published:** 2015-06-08

**Authors:** Hao Wang, Richard D. Robinson, John S. Garrett, Kellie Bunch, Charles A. Huggins, Katherine Watson, Joni Daniels, Brett Banks, James P. D'Etienne, Nestor R. Zenarosa

**Affiliations:** ^1^Department of Emergency Medicine, Integrative Emergency Services Physician Group, John Peter Smith Health Network, 1500 S. Main Street, Fort Worth, TX 76104, USA; ^2^Department of Emergency Medicine, Baylor University Medical Center, 3501 Junius Street, Dallas, TX 75246, USA

## Abstract

*Background*. The accuracy and utility of current Emergency Department (ED) crowding estimation tools remain uncertain in EDs with high annual volumes. We aimed at deriving a more accurate tool to evaluate overcrowding in a high volume ED setting and determine the association between ED overcrowding and patient care outcomes.* Methods*. A novel scoring tool (SONET: Severely overcrowded-Overcrowded-Not overcrowded Estimation Tool) was developed and validated in two EDs with both annual volumes exceeding 100,000. Patient care outcomes including the number of left without being seen (LWBS) patients, average length of ED stay, ED 72-hour returns, and mortality were compared under the different crowding statuses.* Results*. The total number of ED patients, the number of mechanically ventilated patients, and patient acuity levels were independent risk factors affecting ED overcrowding. SONET was derived and found to better differentiate severely overcrowded, overcrowded, and not overcrowded statuses with similar results validated externally. In addition, SONET scores correlated with increased length of ED stay, number of LWBS patients, and ED 72-hour returns.* Conclusions*. SONET might be a better fit to determine high volume ED overcrowding. ED overcrowding negatively impacts patient care operations and often produces poor patient perceptions of standardized care delivery.

## 1. Introduction

Emergency Department (ED) overcrowding has become more and more prevalent throughout the nation in recent years, especially in the high volume ED settings [1–3]. In previous studies, severe overcrowding was reportedly linked to insufficient ED operations management which negatively affect patient care outcomes, including increased length of stay (LOS) at ED, number of patients who eloped or left without being seen (LWBS), ED/hospital mortality, patients that returned to ED within 72 hours of discharge, ambulance diversions, and medical errors [4–6]. It is important to accurately determine ED overcrowding thereby providing administrators' meaningful tools to properly manage ED flow. Historically different ED overcrowding tools have been used in different ED settings with inconsistent accuracy and the association between ED overcrowding and negative patient outcomes is noted to vary accordingly [7–11].

Different ED crowding report systems were also used with different ED overcrowding estimation tools. Some were very complicated and used too many categories (i.e., not busy, busy, crowded, overcrowded, and severely overcrowded), while others were too simple providing only crowded versus not crowded statuses [7–11]. The main purpose for any given ED overcrowding estimation tool should be emphasized by its ability to (1) alert administrators/managers when the ED is approaching maximum capacity and (2) determine when immediate action is needed to prevent conversion to a dysfunctional status. It is more intuitive to estimate ED crowding via a “traffic light system” model that employs only three statuses to determine: (1) green (ED at normal functional status), (2) yellow (ED at alert status), and (3) red (ED approaching and/or existing in a dysfunctional status) [12].

Noting increased population in some regions accompanied with limited healthcare resources, EDs in these areas tend to commonly see extremely high volumes (>100,000 annually) thereby resulting in more frequent and severe ED overcrowding. The National Emergency Department Overcrowding Study (NEDOCS) scoring tool is by far one of the most commonly used overcrowding determination adjuncts used throughout the nation with reports of relatively high consistency. However, a study on overcrowding found that using the NEDOCS scale cannot accurately determine overcrowding status in an extremely high volume ED setting [13].

The primary goal of this study is to derive and validate a more accurate tool we named SONET (Severely overcrowded-Overcrowded-Not overcrowded Estimation Tool) to evaluate overcrowding in an extremely high volume ED setting. This overcrowding estimation tool is simplified to only three categories which match the ED “traffic light system” model. Our secondary goal is to determine the association between ED overcrowding and negative patient care outcomes.

## 2. Materials and Methods

### 2.1. Study Design and Sample Size Estimation

This was a prospective study designed to determine overcrowding status in an extremely high volume ED setting with one center deriving an overcrowding estimation tool and the other center validating it. This study was initially carried out at a publicly funded hospital whose ED is an academic department supporting an Emergency Medicine residency program. The annual volume of the study ED is over 113,000. Additionally this study is also intended to compare the accuracy of the NEDOCS scoring tool in this clinical environment. The John Peter Smith Health Network institutional review board approved the study (IRB approval number: 110413.003ex) with the waiver of informed consent due to the absence of a personal health information requirement and analysis of unidentified data. In addition, an external validation study was also carried out in a different city at a community hospital ED with an annual volume of over 111,000.

Sample size was estimated on the basis of our previous ED crowding estimation data using NEDOCS scales. The previous study was conducted in June of 2013. The results of our previous study demonstrated NEDOCS inaccuracy in determining ED overcrowding [13]. In our setting, NEDOCS scores fell into overcrowded status more than 75% of the times. Average length of stay demonstrated no statistically significant difference among patients registered under different crowding conditions [13]. We conducted a modified Delphi survey including the lead physicians, nursing team leaders, and flow coordinators to determine the level of ED overcrowding beyond which ED operational efficiency begins to decline. Our survey results showed that ED flow could reasonably be expected to be maintained at an overcrowding level of approximately 60%. The comparison study designed to determine the accuracy of ED crowding estimation by both NEDOCS and SONET was thus developed. Setting the acceptable significance level (*α* = 0.01) at 99% for two-tailed alternative hypothesis and assigning the power of the study at 80% (*β* = 0.2), we estimated the sample size to be 222 different time points by using the formula *N* = (*Z*_*α*/2_ + *Z*_1−*β*_)^2^ × (*p*_1_ × (1 − *p*_1_) + *p*_2_ × (1 − *p*_2_))/(*p*_1_ − *p*_2_)^2^, where *p*_1_ is defined as 75% of overcrowding status as determined by NEDOCS and *p*_2_ is defined as 60% of overcrowding status as determined by SONET. *Z*_*α*/2_ is the normal deviation at a level of 99% significance and *Z*_1−*β*_ is the normal deviation at 1 − *β*% power with 20% of a type II error [14]. In addition, with the expected attrition rate of 10%, the final estimate sample size is 244 time points needed to determine a significant difference between the NEDOCS and SONET groups. With 12 different time points measured within one day, it was determined that a 21-day trial with a total of 252 different time point measurements is required for this study.

### 2.2. Operational Variables

Operational variables that were used to derive the NEDOCS score were also included in this study, such as the total number of patients in the ED, total admission holds in the ED, number of ventilators used in the ED, longest admission hold time (in hours), and longest waiting room time (in hours) of the most recent patient placed in a bed in the ED. Two constants including total ED beds and total hospital beds were also recorded. In this study, we collected these operational variables to calculate the NEDOCS score. Other clinical or operational variables such as the number of attending physicians, nurses, and residents on duty, the number of patients with different patient acuity levels, and the longest wait time of those patients in the waiting room at the time of scoring were also collected.

In addition, the Total Emergency Severity Index (hereafter referred to as TESI) was also calculated and considered as a potentially reliable discreet data element affecting ED overcrowding [8]. The index was calculated using the reversal of the acuity levels at triage for patients who were placed in an ED bed and seen by an ED physician/resident. This calculation is defined as TESI = ∑*n*_*i*_ *t*_*i*_/*N*, where *n*_*i*_ indicates the number of patients with acuity level category *i*, *t*_*i*_ indicates the reverse of the standard acuity level category (ordinal scale of 1–5 with 1 being the least acute patients and 5 being the most acute patients), and *N* indicates the total number of patients. Besides TESI, other indexes were generated including total numbers of patients, physicians, and nurses. The total patient index is the total number of patients in the ED divided by the number of ED beds. The physician index is the total number of patients in the ED divided by the number of physicians on duty. The nurse index is the total number of patients in the ED divided by the number of nurses on duty.

### 2.3. Study Protocol and Outcome Measurement

The derivation study was carried out from July 15, 2013, through August 5, 2013. During these 21 days, the NEDOCS score was calculated by using an online NEDOCS score calculator (http://www.nedocs.org/) every two hours. At the same time, all physicians, charge nurses, flow coordinator nurses, and residents were called separately and asked to report their perception of the current ED overcrowding status. The perceptions of ED overcrowding were rated on a 0–10 cm visual analogue scale (VAS). ED overcrowding was considered if the score on the VAS ≥ 5 and considered severely overcrowded if the score on the VAS ≥ 7 which was used as the same scale in other ED overcrowding studies [9, 11]. An average ED overcrowding score was then calculated. In order to confidently compare to NEDOCS scores, it was multiplied by a factor of 20. In addition, all variables mentioned above were recorded simultaneously by a dedicated clerk who did not participate in this study. A scoring tool (SONET) to determine ED overcrowding was then derived from the study and further compared with the NEDOCS score. Additionally, an internal validation was performed using the same data set.

All patients during the study period were assigned to have NEDOCS scores calculated at the time the patients were registered in the ED and stratified into three different overcrowding categories. Patients that were directly admitted by other services and immediately moved out of the ED were excluded from this study. Patients that were transferred to the ED from the on-site urgent care center and requiring a higher level of care were considered as potential high risk presentations and were excluded from the study because these patients received priority ED bed placement regardless of their individual acuity level based on hospital policy. Patients who registered at the ED with no NEDOCS scores calculated during the study time period due to incomplete data were also excluded from this study. SONET score was derived after the study was completed and retrospectively entered into the study data to compare with the NEDOCS score of the same patient.

In order to determine whether ED overcrowding affects ED operations, ED LOS and the number of LWBS patients, ED/hospital mortality, and returns to ED within 72 hours were used as markers for ED efficiency measurements. ED/hospital mortality refers to a patient's death when that patient was still physically in the ED or had been initially admitted to the hospital from the ED. Patients that returned to the ED within 72 hours of initial service were analyzed based on their chief complaint, history of present illness, and admission/discharge diagnosis as recorded for the initial visit. All patients registered for ED services during the study period were included in the data analysis. Those patients that returned to the ED within 72 hours of initial presentation were reviewed by two independent physicians who were blinded to this study. Patients who had the same chief complaint or any complaint that might be directly related to the first ED visit were considered ED return patients. Any discrepancy between the two reviewing physicians was further reviewed and resolved by a third physician to obtain general consensus. Additionally, all these operational outcomes measurements were analyzed and compared in different ED overcrowding conditions as determined by the NEDOCS scale.

### 2.4. External Validation Study

An external validation study was carried out from 8 am on August 6 to 6 am on August 27, 2014. During these 21 days, the NEDOCS score was again calculated every two hours. At the same time point, a SONET score was calculated as well. All patients during the study period were assigned to three different overcrowding categories (e.g., not overcrowded, overcrowded, and severely overcrowded groups) based upon the scores calculated by NEDOCS and SONET. Patients who registered at the ED with neither NEDOCS nor SONET scores calculated during the study time period due to incomplete data were excluded from this validation study. Patient general characteristics and ED operational variables (see detail in Table 1, right panel) were compared with the derivation study. In addition, patients average LOS at ED and their LOS under the different acuity levels were measured and compared under the different overcrowding conditions determined by NEDOCS and SONET scores. The Baylor University Medical Center institutional review board approved the study (IRB approval number: 14-013).

### 2.5. Data Analysis and Statistics

A linear regression model was applied and the independent operational variables that could affect ED overcrowding status scores were determined. Adjusted *R*-square was used to determine the power of the model fitting the data. Correlation coefficiency (*r*) was analyzed on each operational variable with its scatter plot drawn. Variables that had strong correlation with ED overcrowding were chosen for linear regression analysis. Variance inflation factor (VIF) quantifies the severity of multicollinearity in the regression model analysis thereby providing an index to estimate whether the regression coefficient is increased due to collinearity. Operational variables with high VIF (>10) were considered as having collinearity and were therefore excluded from regression analysis [15, 16]. A formula was then generated based on the regression coefficient of each independent operational variable and an ED overcrowding score was calculated. The SONET score calculated from this study was also compared with the NEDOCS score. Receiver operational characteristic (ROC) curves were drawn and areas under the ROC curve (AUC) were measured and compared between SONET and NEDOCS scores. A bootstrap technique that randomized 1,000 samples was used to internally validate the study score accuracy.

Considering the operational significance of determining ED overcrowding status, the SONET score was divided into three categories: not overcrowded (score < 100), overcrowded (score between 100 and 140, including 100 but not including 140), and severely overcrowded (score ≥ 140). Patients were automatically assigned to three groups based on ED overcrowding scores at the time when a specific patient registered for services in the ED. To compare the differences between LWBS, ED/hospital mortality, and returns to ED within 72 hours relative to the different ED overcrowding status groups, analysis of variance (ANOVA) with Bonferroni correction was used to analyze differences between groups.

All statistical analysis was performed using STATA 12 (College Station, TX) and a *p* < 0.05 was considered a statistically significant difference.

## 3. Results

NEDCOS scores might overestimate ED severely overcrowded status. A new ED overcrowding scoring tool (SONET) that demonstrates a relatively more accurate and reliable estimation of ED overcrowding was derived.

**Figure 1:**
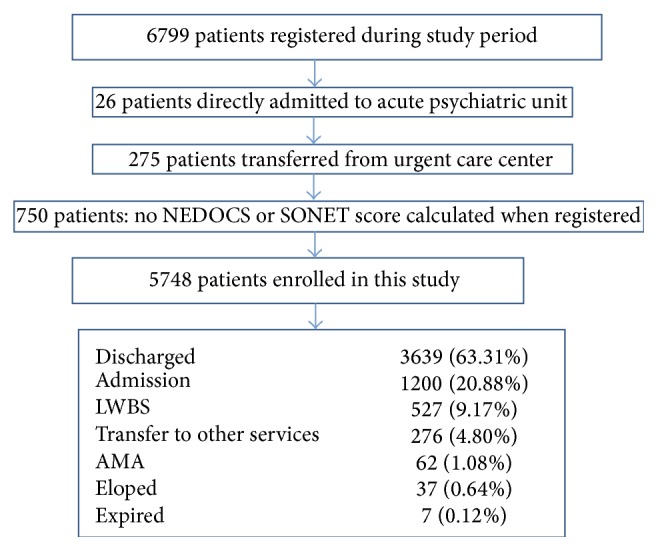
It shows the flow diagram of patient included in the SONET derivation study.

The prospective derivation study was performed from 8 am on July 15, 2013, until 6 am on August 5, 2013. During this 21-day period all operational variables from NEDOCS were recorded and scores were calculated every two hours. A total of 6,799 patients were registered to receive services with the final of 5,748 patients enrolled during the study period (Figure 1 and Table 1). At the same time, other SONET specific test variables were also collected. There were 206 data sets collected at different time points resulting in a data completion rate of 81.7% (206/252). When tied to the three different crowding statuses (not overcrowded, overcrowded, and severely overcrowded), statistically significant differences were noted when comparing ED overcrowding status between the SONET and the NEDOCS scores (Table 2, *p* < 0.001). In addition, the interrater reliability between the NEDOCS and the SONET scores in determining ED overcrowding status was weak (*κ* = 0.3811, *p* < 0.001).

Results of linear regression demonstrated 4 variables that can be considered independent risk factors affecting ED overcrowding status. The adjusted *R*-square was 0.7872 indicating a strong power of prediction in this regression model. Other variables reached no statistical significance, had no correlation with overcrowding, or had significant collinearity with a VIF (variance inflation factor) greater than 10. Therefore a new ED overcrowding scoring formula (SONET) was derived and is defined as follows.

SONET score = 0.8 × number of acuity level-3 patients in the waiting room + 0.5 × number of acuity level-2 patients occupying an ED bed + 10 × number of ventilation patients in the ED + 53 × total patient index + 18. (In short form: SONET score = 0.8*W* + 0.5*A* + 10*V* + 53*E* + 18, where *W* indicates the number of acuity level-3 patients in the waiting room, *A* indicates the number of acuity level-2 patients occupying an ED bed, *V* indicates the number of patients on ventilators in the ED, and *E* indicates the total number of ED patients divided by the total number of ED beds. An extremely short form is “WAVE.”) A SONET score ≥ 100 is considered the threshold for ED overcrowded status and ≥140 is considered the threshold for severely overcrowded status.

Using the average perceptions of ED overcrowding among the different healthcare providers as a “gold standard,” ROC curves were drawn between SONET and NEDOCS scores. The results showed greater accuracy in predicting ED overcrowded status by the SONET score (AUC = 0.9568, 95% CI 0.9334–0.9802) than the NEDOCS score (AUC = 0.9202, 95% CI 0.8797–0.9607, *p* = 0.0389). Similar results occurred in predicting ED severely overcrowded status by the SONET (AUC of the SONET 0.9307, 95% CI 0.8941–0.9672 versus AUC of the NEDOCS 0.8902, 95% CI 0.8436–0.9367, *p* = 0.0292). Internal validation using the bootstrap method yielded similar results (data not shown).

ED overcrowding status was associated with increased LOS at ED, the number of LWBS patients, and the numbers of patients returning to the ED within 72 hours. However, ED/hospital mortality numbers were not affected by ED overcrowding status.

The average LOS in ED was analyzed under the different overcrowding status scores as determined by NEDOCS and SONET separately. The average LOS in ED under different overcrowding status scores determined by SONET reached a statistically significant difference between groups, especially when compared with groups of different acuity levels (hereafter referred to as emergency severity index (ESI) level, see Table 3). The more severely overcrowded the ED, the longer the average LOS of all patients, especially under the ESI-3, ESI-4, and ESI-5 categories. When analyzing only discharged patients, similar results occurred with a statistically significant difference among groups (Table 3). When ED overcrowding status was determined by NEDOCS, it showed similar results but was not as significantly differentiated as that seen with SONET (Table 4).

LWBS data was collected every two hours. Results showed that the numbers of LWBS patients were associated with the severity of ED overcrowding as determined by both SONET and NEDOCS scores. However, no statistically significant difference was found between the not overcrowded versus overcrowded conditions determined by the NEDOCS score (Table 5).

During the study period, 7 patients died in the ED and 23 patients died in the hospital after ED admission. All 7 patients that died while in the ED were directly related to trauma and all were brought in by emergency medical service (EMS) personnel. These patients were seen immediately by ED physicians and trauma surgeons. Of the patients that died in the hospital after ED admission, 19 of 23 were brought to the ED either by EMS or by private car and determined to have a status of do not resuscitate (DNR) due to various end stage diseases. The remaining 4 patients presented to the ED via EMS with no DNR status (Table 6). Study results showed no strong association between mortality and ED overcrowding by using either NEDOCS or SONET scores (*p* > 0.05). For this subset of patients the ED overcrowding status indicated by both NEDOCS and SONET scores showed a reasonable level of agreement (*κ* = 0.6, *p* = 0.0352).

**Figure 2:**
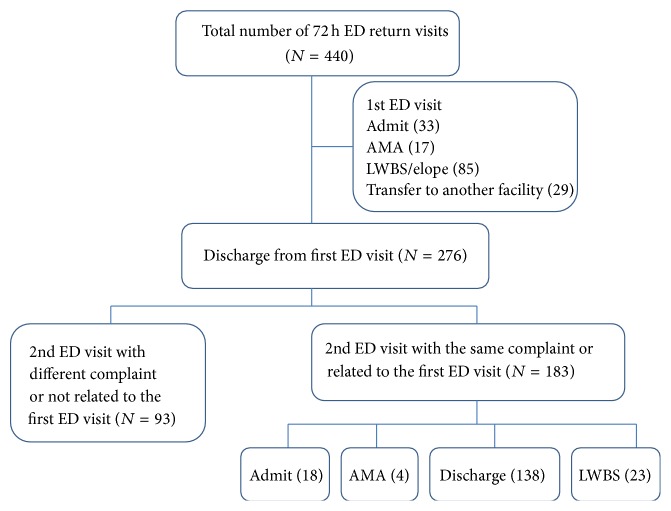
It shows the flow diagram of patients returning to the ED within 72 hours of initial visit during the derivation study period. There were total 440 ED returns in which 276 patients were discharged at their first ED visit. 66.3% (183/276) presented within 72 hours of their initial ED visit with the same complaint or one directly related to the initial one. Of these 9.8% (18/183) were admitted to hospital at their second ED visit. Follow-up of these patients in hospital showed no significant worsening of their outcomes.

A total of 440 patients returned to the ED within 72 hours of an initial visit during the study period. Only 183 of these patients made ED returns that were directly related to the initial ED visits. Of those returning, 18 were admitted initially and then discharged with no bad outcome (Figure 2). Both the NEDOCS and SONET scores of all 18 patients at their first ED visit showed a status of either overcrowded or severely overcrowded, indicating ED overcrowding was strongly associated with 72-hour ED returns.

An external validation study confirmed the more accurate use of SONET scores in an extremely high volume ED setting.

The external validation study was also done in a 21-day period at a community ED of extremely high volume. During these 21 days, data were collected every 2 hours. There were a total of 232 completed data sets collected at different time points with a data completion rate of 92.06% (232/252). The results were different than those seen at JPS regarding the percentage of overcrowded status (Table 2, right panel). A similar trend occurred when comparing overcrowding statuses as determined by NEDOCS (Table 3).

Similarities were noted in terms of general patient characteristics (e.g., gender and age) and operational variables (including numbers of patients, ESI levels, and admission rates) when comparing the derivation and validation study sites (Table 1). However, the average LOS at ED under different overcrowding statuses was different when considering whether the overcrowding status was determined by NEDOCS versus SONET (Table 7). It appears that no trend developed in terms of prolonged ED stays when comparing the different overcrowding statuses scored by NEDOCS. However, a trend is noted when comparing prolonged ED stays associated with different overcrowding statuses scored by SONET although no significant statistical difference is appreciated. Further analysis of the LOS of patients with different acuity levels demonstrated similar results (Table 8). This confirmed the improved accuracy of SONET in determining the relative overcrowding status in an extremely high volume ED setting.

## 4. Discussion

NEDOCS is currently considered the most commonly used ED overcrowding scoring tool developed to date. Uncertainty exists in regard to its accuracy when determining the relative degree of ED overcrowding status across diverse clinical environments [7, 17, 18]. It was developed in academic EDs with moderate to high annual volumes. Our study was carried out at a publicly funded hospital whose ED is an academic department supporting an Emergency Medicine residency program. Results of our previous study showed using NEDOCS to determine overcrowding status might not be reliable in an extremely high volume ED setting [13]. Therefore, in order to determine the accuracy and consistency of overcrowding scoring tools in an extremely high volume ED setting, SONET was derived and compared to NEDOCS.

One of the most common models used to estimate ED overcrowding status was based on the “input-throughput-output” theory and is related to multiple independent operational factors [19–21]. The numbers of patients in the waiting room along with those arriving by ambulance are considered input factors which are typically uncontrolled variables. The illness severity of patients in the ED, the numbers of adjunct exams and procedures performed in the ED, and the numbers of physicians and nurses on duty significantly affect ED throughput times. Output factors such as movement of admitted, transferred, and boarded patients in the ED also affect efficiency. These operational variables may act as independent risk factors or produce synergistic effects while others may be confounders. Therefore efficient ED management in the setting of ED overcrowding requires a means to accurately and reliably predict a relative overcrowding status that may trigger near real-time implementation of predetermined operational interventions designed to mitigate the negative effects of overcrowding with respect to quality healthcare delivery and patient and staff experiences.

Several ED overcrowding estimation tools were derived using different operational parameters. The Emergency Department Work Index (EDWIN) included the total number of patients in the ED, the illness severity of those patients, the total number of ED beds, and the number of patients boarded in the ED as operational parameters to develop a formula for estimating ED overcrowding status [11]. The ED Work Score found that the number of patients in the waiting room, the illness severity of those patients, the number of total ED beds, and the numbers of nurses on duty affected ED crowding status [10]. The ED Occupancy Rate was calculated from the number of patients in the ED and the number of ED beds [8]. The NEDOCS is one tool that is used commonly and in different ED settings nationwide [7]. It is limited to five variables using a mathematical formula but does not include physician and nurse staffing levels and patient illness severity level. Apart from population selection bias, these ED overcrowding estimation tools have limitations and none of them include all of the potential operational parameters together in a single tool [9]. Our study included 20 different operational parameters that we can collect and analyze together to determine an estimate of ED overcrowding. In our study, 4 out of 20 operational parameters were considered independent predictors with no collinearity. The study score also showed better accuracy compared with the NEDOCS score indicating greater confidence in estimation of ED overcrowding status in the study population.

The utility of a given tool remains its ability to effectively communicate the relative overcrowding status of the ED in near real time and provide an accurate prediction of additional resource needs to clinical staff and administration in order to provide for a safe environment. Our ED overcrowding estimation scoring tool, Severely overcrowded-Overcrowded-Not overcrowded Estimation Tool (SONET), was derived in the setting of a publicly funded tertiary care hospital with an Emergency Medicine residency program and much higher annual volumes than those used to derive NEDOCS. SONET was then externally validated in a community ED with similar annual volumes. A SONET score of not overcrowded indicates a status within which the ED functions properly. When SONET produces an overcrowded score it indicates the ED is at maximum capacity and administrators should closely monitor ED flow and begin to mobilize additional resources. When SONET produces a score of severely overcrowded additional resources must be immediately deployed to decompress ED congestion. When linked to operational management, it is considered more intuitive to report under the “severely overcrowded (red), overcrowded (yellow), and not overcrowded (green)” categories, similar to a “traffic light system” [12]. Taken together, SONET may ultimately be found to be more reliably applied in a similar ED setting.

ED overcrowding is linked to negative patient care outcomes such as increased LWBS rates and ED LOS [22–25]. These negative patient care outcomes can be improved through interventions designed to decrease ED overcrowding [26–28]. Our study confirmed that the average ED LOS is associated with ED overcrowding and demonstrates high correlation when a status of severely overcrowded is reached and sustained. However, it is limited to only discharged patients with initial ESI level of 3–5 because patients with ESI level of 1-2 are considered high risk patients requiring immediate intervention regardless of relative ED overcrowding status. In addition, the majority of these high risk patients in the study ED arrived by ambulance with EMS reports provided while en route to the hospital thereby resulting in immediate bed placement upon presentation. In the subset of patients admitted to hospital from the ED we observed a trend of prolonged LOS in all the patients when the ED became more overcrowded although no statistically significant difference was reached especially within patients triaged to ESI levels of 1, 4, and 5 (data not shown). ED overcrowding may partially be attributed to LOS yet other operational variables affecting overall hospital crowding play important roles in the subset of admitted patients [29, 30]. There were only 28 ESI level 4-5 patients admitted to hospital during the study thereby negatively impacting statistical power for analysis. As mentioned above, patients triaged as ESI-1 are considered critically ill patients and therefore receive immediate attention upon arrival. ED overcrowding has no significant effect on these patients as they typically move through the ED and into the inpatient setting with minimal to no delay.

Our study found that the LWBS rate is associated with ED overcrowding and demonstrates high correlation when a status of severely overcrowded is reached and sustained. This was also consistent with the reports of previous studies [31, 32]. A significant admission rate was noted among the subset of patients that returned to the ED within 72 hours of initial visit. Most of these patients were seen while the ED was in an overcrowded status during their initial visits. No adverse medical outcomes were noted among these patients. The majority of patients in the LWBS and return to ED within 72-hour subsets were initially triaged as ESI-3 level patients (urgent but not emergent status). ED overcrowding status does not appear to affect ED/hospital mortality rates as these patients were all seen by a physician immediately upon arrival and all were triaged as ESI-1 level patients (emergent status). This may suggest that acuity level plays an important role in patient care operations when the status of ED overcrowded is reached. This also suggests that ESI-3 level patients are the subset whose care outcomes could be significantly affected under the different ED overcrowding statuses.

Overall, our study outcomes indicate SONET might be a suitable tool to determine ED overcrowding in an extremely high volume ED. In addition, ED overcrowding is linked to negative patient care outcomes including increased average ED LOS and number of LWBS patients.

*Limitations*. The SONET tool was derived from a single urban academic ED affiliated with a publicly funded hospital system which has a very high annual ED volume. Results may be skewed due to selection bias associated with the study population. Considering these data were from a single institution, we performed an internal validation using data randomized from the same study by bootstrap and an external validation in a community ED of similar volume. Data analyses showed consistent results indicating the reliability of using the SONET tool to determine ED overcrowding status. As mentioned above, we realize that the study results may be skewed by virtue of population selection. Therefore, an even larger multicenter study among similar ED environments is required to achieve extensive external validation.

At present, there remains no gold standard tool capable of defining ED overcrowding. Sole reliance on perceptions of different healthcare providers may be overly subjective. However, a previous study tested the interrater and intrarater variability of healthcare providers' perceptions and results demonstrated a moderate to good agreement across study participants [13]. The SONET tool was derived based on the average level of perceptions of ED overcrowding by the same group of healthcare providers which should therefore minimize the bias of individual subjective judgments.

Our incomplete data was relatively higher than we initially estimated (18.3% versus 10%) which resulted in a relatively smaller sample size (206 time points). This is based on the calculated sample size anticipated to reach a 99% significance level. However, it does provide sufficient data to obtain a 95% significance level for statistical analysis. In addition, our realized result of a 60.19% ED overcrowding level was very close to the calculated Delphi estimation of 60%. A similar sample size produced similar results when analyzing the data from the external validation study.

Finally, using the SONET score to determine overcrowding status might only apply to similar setting EDs because perceptions of overcrowding might vary among different healthcare providers working in different ED environments. This tool might not be suitable for a relatively low volume ED setting.

## 5. Conclusion

Overall, our study outcomes indicate SONET might be a better tool to determine overcrowding in an ED setting of extremely high volume. This scoring system is intended to differentiate only severely overcrowded, overcrowded, and not overcrowded statuses. In addition, ED overcrowding can negatively affect patient care operations and often produces poor patient perceptions of standardized care delivery.

## Supplementary Material

The supplementary material includes the checklist detail of using SQUIRE guideline for this manuscript publication. SQUIRE refers to as Standard for Quality Improvement Reporting Excellence. Seventeen items that required by SQUIRE guideline were reported in this paper and listed in detail in the supplementary material.

## Figures and Tables

**Table 1:** General patient characteristic and operational variables in both the derivation and validation studies.

General information	July 2013 study at JPS	Aug 2014 study at BUMC
Total number of patients (*n*)	6,799	6,302
Number of patients for data analysis (*n*)	5,748	6,037
Male (%)	2,620 (45.58%)	2,724 (45.12%)
Age (mean (SD), 95% CI)	(42.48 (15.45), 42.07–42.89)	(44.60 (20.28), 44.09–45.11)
Acuity level (*n*)		
ESI-1	200	60
ESI-2	1,351	1,979
ESI-3	2,560	2,551
ESI-4	1,278	1,249
ESI-5	283	184
Unclassified	76	9
Total number of admissions (%)	1,200 (20.88%)	1,288 (21.34%)
Total number of patients LWBS (%)	527 (9.17%)	296 (4.90%)

ED: Emergency Department; SD: standard deviation; CI: confidence interval; ESI: emergency severity index; LWBS: left without being seen.

In this study, a total of 5,748 patients from JPS had age and gender information due to restricted information not able to be released for other 245 patients.

**Table 2:** Percentage of ED crowding statuses determined by the SONET and NEDOCS scores in both the derivation and validation studies.

Emergency Department	JPS Health Network		Baylor University Medical Center	
	NEDOCS (*N* = 206)	SONET (*N* = 206)	NEDOCS (*N* = 232)	SONET (*N* = 232)
Severely overcrowded	100 (48.54%)	39 (18.93%)	9 (3.88%)	2 (0.86%)
Overcrowded	53 (25.73%)	85 (41.26%)	52 (22.41%)	32 (13.79%)
Not overcrowded	53 (25.73%)	82 (39.81%)	171 (73.71%)	198 (85.34%)

*N* indicates the number of time points that the ED crowding status was measured. JPS ED noted a 61% decrease in severely overcrowded scores when using SONET as compared to NEDOCS tools. The overall overcrowded status scores decreased from 74.27% to 61.19% (13.08%). BUMC ED noted a 77% decrease in severely overcrowded scores when using SONET as compared to NEDOCS tools. The overall overcrowded status scores decreased from 26.29% to 14.66% (11.63%).

**Table 3:** Comparison of the average length of stay in patients with different acuity levels under different ED overcrowding status determined by the SONET score in the derivation study.

ED crowding status	ESI-1 (h) Mean (SD) (*n*)	ESI-2 (h)	ESI-3 (h)	ESI-4 (h)	ESI-5 (h)
Patients that were discharged from ED (number of patients)					
Not overcrowded	5.02 (1.93) (11)	4.77 (2.71) (219)	4.35 (2.47) (659)	3.06 (1.75) (409)	2.37 (1.40) (110)
Overcrowded	5.58 (1.87) (9) (^*∗*^1.000)	4.96 (2.61) (272) (^*∗*^1.000)	6.08 (3.15) (719) (^*∗*^<0.001)	4.25 (2.06) (485) (^*∗*^<0.001)	3.39 (1.43) (82) (^*∗*^<0.001)
Severely overcrowded	4.95 (2.51) (7) (^*∗∗*^1.000)	5.26 (4.13) (132) (^*∗∗*^1.000)	7.05 (3.67) (307) (^*∗∗*^<0.001)	5.12 (2.47) (170) (^*∗∗*^<0.001)	4.52 (2.53) (34) (^*∗∗*^0.002)

All patients that were initially registered at ED (number of patients)					
Not overcrowded	5.21 (5.21) (66)	6.35 (4.28) (477)	5.17 (3.54) (898)	3.30 (2.19) (452)	2.39 (1.41) (122)
Overcrowded	6.35 (6.12) (78) (^*∗*^0.680)	6.87 (5.31) (604) (^*∗*^0.287)	6.90 (4.61) (1132) (^*∗*^<0.001)	4.69 (2.95) (589) (^*∗*^<0.001)	3.81 (2.56) (109) (^*∗*^<0.001)
Severely overcrowded	5.42 (5.23) (56) (^*∗∗*^1.000)	7.66 (5.71) (269) (^*∗∗*^0.102)	7.52 (4.91) (530) (^*∗∗*^0.020)	5.26 (2.68) (237) (^*∗∗*^0.018)	4.90 (2.92) (52) (^*∗∗*^0.012)

ESI: emergency severity index; Mean: the average of length of stay (LOS) in hours; SD: standard deviation; *n*: the number of patients. ^*∗*^ *p* value of the comparison between two groups of patients under the different ED crowding conditions (not overcrowded versus overcrowded). ^*∗∗*^ *p* value of the comparison between two groups of patients under the different ED crowding conditions (overcrowded versus severely overcrowded).

**Table 4:** Comparison of the average length of stay in patients with different acuity levels under different ED overcrowding status determined by the NEDOCS score in the derivation study.

ED crowding status	ESI-1 (h) Mean (SD) (*n*)	ESI-2 (h)	ESI-3 (h)	ESI-4 (h)	ESI-5 (h)
Patients that were discharged from ED (number of patients)					
Not overcrowded	5.58 (1.35) (5)	4.68 (2.46) (130)	4.26 (2.63) (461)	2.99 (1.76) (259)	2.11 (1.36) (76)
Overcrowded	5.19 (2.31) (9) (^*∗*^1.000)	4.72 (2.55) (158) (^*∗*^1.000)	5.19 (2.91) (415) (^*∗*^<0.001)	3.52 (1.69) (314) (^*∗*^0.006)	2.88 (1.17) (53) (^*∗*^0.026)
Severely overcrowded	5.04 (2.21) (13) (^*∗∗*^1.000)	5.17 (3.40) (335) (^*∗∗*^0.386)	6.53 (3.31) (809) (^*∗∗*^<0.001)	4.69 (2.34) (491) (^*∗∗*^<0.001)	3.91 (1.98) (97) (^*∗∗*^0.001)

All patients that were initially registered at ED (number of patients)					
Not overcrowded	5.24 (5.46) (36)	5.86 (3.53) (266)	5.10 (3.70) (623)	3.23 (2.31) (282)	2.26 (1.52) (86)
Overcrowded	5.33 (4.57) (49) (^*∗*^1.000)	6.50 (4.61) (368) (^*∗*^0.338)	6.38 (4.55) (642) (^*∗*^<0.001)	3.82 (2.33) (352) (^*∗*^0.016)	2.97 (1.68) (63) (^*∗*^0.165)
Severely overcrowded	6.02 (6.02) (115) (^*∗∗*^1.000)	7.38 (5.69) (716) (^*∗∗*^0.020)	7.09 (4.55) (1295) (^*∗∗*^0.002)	5.04 (2.94) (644) (^*∗∗*^<0.001)	4.33 (2.78) (134) (^*∗∗*^<0.001)

ESI: emergency severity index; Mean: the average of length of stay (LOS) in hours; SD: standard deviation; *n*: the number of patients. ^*∗*^ *p* value of the comparison between two groups of patients under the different ED crowding conditions (not overcrowded versus overcrowded). ^*∗∗*^ *p* value of the comparison between two groups of patients under the different ED crowding conditions (overcrowded versus severely overcrowded).

**Table 5:** Association between ED crowding and LWBS patients using the different scoring systems in the derivation study. The number of LWBS patients increased in proportion to the severity of ED crowding as determined by both NEDOCS and SONET scores when comparing all three categories (*p* < 0.001). However, the number of LWBS patients was higher in both overcrowded and severely overcrowded statuses determined by the SONET but not the NEDOCS. This study showed a positive association between increasing numbers of LWBS patients as a function of ED crowding levels.

ED crowding status estimation	Number of LWBS patients every two hours (*n*)	
	NEDOCS score	SONET score
Not overcrowded	0.5 (1.0)	0.7 (1.4)
Overcrowded	1.7 (1.8), (^*∗*^0.106)	3.1 (2.8), (^*∗*^<0.001)
Severely overcrowded	4.1 (3.7), (^*∗∗*^<0.001)	5.3 (4.4), (^*∗∗*^<0.001)

LWBS: left without being seen; *n*: the number of patients. ^*∗*^ *p* value of the comparison between two groups of patients under the different ED crowding conditions (not overcrowded versus overcrowded). ^*∗∗*^ *p* value of the comparison between two groups of patients under the different ED crowding conditions (overcrowded versus severely overcrowded).

**Table 6:** General information on patients who were not initially identified as DNR and who ultimately died in hospital in the derivation study. There were a total of 4 patients that died within the study period. They were all transported via ambulance. NEDOCS and SONET scores were assigned to each patient at the time when they registered in the ED. Two of these four patients were registered in the ED during a time when the environment was considered severely overcrowded. However both patients were immediately seen by physicians and residents due to the severity of their illness. The average length of stay in the ED was 41 ± 11.6 minutes. Due to the limited number of patients, our results showed no association between ED crowding and mortality.

Patient number	Chief complaint	MOA	ED LOS	Total LOH	NEDOCS score^*∗*^	SONET score^*∗*^
1	Cardiac arrest	EMS	25 min	8 h, 20 min	Overcrowded (103)	Not overcrowded (67)

2	Acute respiratory distress	EMS	40 min	6 days	Severely overcrowded (142)	Severely overcrowded (144)

3	Acute respiratory distress	EMS	48 min	15 h, 58 m	Not overcrowded (85)	Not overcrowded (80)

4	Transfer: gunshot wound to the head	EMS	51 min	10 h, 48 m	Severely overcrowded (143)	Severely overcrowded (160)

MOA: mode of arrival; LOS: length of stay; LOH: length of hospitalization.

^*∗*^The numbers in these two columns stand for the ED crowding score using either NEDOCS or SONET scoring system at the time the individual patient was registered at ED.

**Table 7:** Comparison of the average length of stay in patients under different ED overcrowding statuses by the SONET and NEDOCS in the validation study.

ED crowding status estimation	Average LOS of ED patients at BUMC (*n* = 6,307)		
	NEDOCS score Min (SD) (*n*)	SONET score Min (SD) (*n*)	*p* value
Not overcrowded	264 (179), (5,249)	264 (178), (5,560)	1.000
Overcrowded	278 (187), (700, ^*∗*^0.142)	278 (205), (447, ^*∗*^0.324)	1.000
Severely overcrowded	250 (200), (88, ^*∗∗*^0.509)	292 (178), (30, ^*∗∗*^1.000)	0.3098

BUMC: Baylor University Medical Center; LOS: length of stay at ED; *n*: the number of patients; SD: standard deviation; *p* value: *p* value of the comparison of average LOS between NEDOCS and SONET scores. ^*∗*^ *p* value of the comparison between two groups of patients under the different ED crowding conditions estimated by the same scoring system (not overcrowded versus overcrowded). ^*∗∗*^ *p* value of the comparison between two groups of patients under the different ED crowding conditions estimated by the same scoring system (overcrowded versus severely overcrowded).

**Table 8:** Comparison of the average length of stay in patients with ESI-3 and ESI-4 under different ED overcrowding statuses by the SONET and NEDOCS in the validation study.

ED crowding status	SONET		NEDOCS	
	ESI-3	ESI-4	ESI-3	ESI-4
Not overcrowded (min, *n*) Mean (SD)	286 (161), 2365	157 (106), 1158	286 (161), 2233	158 (108), 1094

Overcrowded (min, *n*) Mean (SD)	302 (235), 176 (^*∗*^1.000)	164 (133), 88 (^*∗*^1.000)	304 (208), 283 (^*∗*^0.498)	159 (114), 135 (^*∗*^1.000)

Severely overcrowded (min, *n*) Mean (SD)	366 (203), 10 (^*∗∗*^1.000)	175 (88), 3 (^*∗∗*^1.000)	287 (199), 35 (^*∗∗*^1.000)	133 (80), 20 (^*∗∗*^1.000)

BUMC: Baylor University Medical Center; ESI: emergency severity index; Mean: the average of length of stay in minutes; SD: standard deviation; *n*: the number of patients. ^*∗*^ *p* value of the comparison between two groups of patients under the different ED crowding conditions estimated by the same tool (not overcrowded versus overcrowded). ^*∗∗*^ *p* value of the comparison between two groups of patients under the different ED crowding conditions estimated by the same tool (overcrowded versus severely overcrowded).
