# Supplementary material for: Use of the SONET Score to Evaluate High Volume Emergency Department Overcrowding: A Prospective Derivation and Validation Study
Source: Emerg Med Int. 2015 Jun 8;2015:401757. doi: 10.1155/2015/401757 (PMC4475699; doi:10.1155/2015/401757)
Supplement: Supplementary file 1 — The supplementary material includes the checklist detail of using SQUIRE guideline for this manuscript publication. SQUIRE refers to as Standard for Quality Improvement Reporting Excellence. Seventeen items that required by SQUIRE guideline were reported in this paper and listed in detail in the supplementary material. [file 401757.f1.pdf]

# Standards for Quality Improvement Reporting Excellence

## SQUIRE guidelines checklist of this manuscript

| Item | Description | Item Detail Explanation | |
|---|---|---|---|
| 1. Title | *a)* Indicates the article concerns the improvement of quality (broadly defined to include the safety, effectiveness, patient centeredness, timeliness, efficiency, and equity of care)<br>*(b)* States the specific aim of the intervention<br>*(c)* Specifies the study method used—for example, qualitative study or randomised cluster trial | a) This is by far, to the best of our knowledge, the first study reported to estimate high volume ED crowding, therefore, no study can be compared.<br>b) The aim of this study is clearly addressed in the title: using SONET score to determine ED overcrowding in a high volume ED setting<br>c) A prospective study | a) N/A<br>b)Yes<br>c)Yes |
| 2. Abstract | Summarises precisely all key information from various sections of the text using the abstract format of the intended publication | Yes, all key information addressed in the abstract including background, methods, results, and inclusions. | Yes |
| 3. Background knowledge | Provides a brief, non-selective summary of current knowledge of the care problem being investigated and characteristics of organisations in which it occurs | Introduction section addressed the current status of ED overcrowding and its need for estimating overcrowding accurately. See Line 54-62, 72-79 | Yes |
| 4. Local problem | Describes the nature and severity of the specific local problem or system dysfunction that was investigated | Addressed in our introduction section (line 72-79) | Yes |
| 5. Intended improvement | *a)* Describes the specific aim (changes/improvements in care processes and patient outcomes) of the proposed intervention<br>*b)* Specifies who (champions, supporters) and what (events, observations) triggered the decision to make changes and why now (timing) | This new ED overcrowding tool was compared with NEDOCS tool<br>Determine the different ED setting triggered the decision to make changes. | Yes (line 63-75) |
| 6. Study question | States precisely the primary improvement related question and any secondary questions that the study of the intervention was designed to answer | The primary and secondary goal were address in the end of the introduction | Yes, Line 80-84 |
| 7. Ethical issues | Describes ethical aspects of implementing and studying the improvement, such as privacy concerns, protection of participants' physical wellbeing, and potential author conflicts of interest, and how ethical concerns were addressed | Line 92-94 | Yes |
| 8. Setting | Specifies how elements of the local care environment considered most likely to influence | Consider different variables might change the estimation accuracy of ED crowding (Line | Yes |

| | | | |
|---|---|---|---|
| | change/improvement in the involved site or sites were identified and characterised | 116-134), consider the different ED setting as mentioned in the Methods and Introduction | |
| 9. Planning the intervention | *a)* Describes the intervention and its component parts in sufficient detail that others could reproduce it<br>*b)* Indicates main factors that contributed to choice of the specific intervention—eg, analysis of causes of dysfunction, matching relevant improvement experience of others with the local situation<br>*c)* Outlines initial plans for how the intervention was to be implemented—eg, what was to be done (initial steps, functions to be accomplished by those steps, how tests of change would be used to modify intervention) and by whom (intended roles, qualifications, and training of staff) | a) the intervention: different ED setting (Line 137-147)<br>b) different operational variables and patient population selection (Line 116-134)<br>c)indication the different variables were obtained during the whole study period (Line 117-124, 148-168) | Yes |
| 10. Planning the study of the intervention | *a)* Outlines plans for assessing how well the intervention was implemented (dose or intensity of exposure)<br>*b)* Describes mechanisms by which intervention components were expected to cause changes and plans for testing whether those mechanisms were effective<br>*c)* Identifies the study design (eg, observational, quasi-experimental, experimental) chosen for measuring impact of the intervention on primary and secondary outcomes, if applicable<br>*d)* Explains plans for implementing essential aspects of the chosen study design, as described in publication guidelines for specific designs,<br>*e)* Describes aspects of the study design that specifically concerned internal validity (integrity of the data) and external validity (generalisability) | a) obtain all variables in the study period and compared with the NEDOCS variables (Line 137-147)<br>b)determine the Delphi technique to reach to an ideal overcrowding model and determine the outcome measurements (Line 98-104)<br>c)identified the study design and determine the outcome measurements (Line 88-96, 157-168)<br>d) report derivation and external validation (Line 88-96)<br>e) report internal and external validation (Line 146-147, 170-180) | Yes |
| 11. Methods of evaluation | *a)* Describes instruments and procedures (qualitative, quantitative, or mixed) used to assess the effectiveness of implementation; the contributions of intervention components and context factors to effectiveness of the intervention; and primary and secondary outcomes<br>*b)* Reports efforts to validate and | a) describe the qualitative study, comparison, primary, and secondary goal, and outcome measurement (see detail in Methods)<br>b) the test reliability and inter-intra-rater variability were reported (Line 140-141)<br>c) this is a single blinded study, persons who collected data | Yes |

| | | | |
|---|---|---|---|
| | test reliability of assessment instruments<br>*c)* Explains methods used to assure data quality and adequacy—eg, blinding, repeating measurements and data extraction, training in data collection, collection of sufficient baseline measurements | received sufficient training on data collection (Line 145-147) | |
| 12.data analysis | *a)* Provides details of qualitative and quantitative (statistical) methods used to draw inferences from the data<br>*b)* Aligns unit of analysis with level at which the intervention was implemented, if applicable<br>*c)* Specifies degree of variability expected in implementation, change expected in primary outcome (effect size), and ability of study design (including size) to detect such effects<br>*d)* Describes analytical methods used to show effects of time as a variable (eg, statistical process control) | a) see detail in statistics section (Line 185-204)<br>b) reported to compare with NEDOCS (Line 193-196)<br>c) mention in sample size estimation (Line 103-114)<br>d)see detail in statistics section (line 185-204) | Yes |
| 13.Outcomes | Nature of setting and improvement intervention:<br>a)Characterises relevant elements of setting or settings (eg, geography, physical resources, organisational culture, history of change efforts) and structures and patterns of care (eg, staffing, leadership) that provided context for the intervention<br>b)Explains the actual course of the intervention (eg, sequence of steps, events, or phases; type and number of participants at key points), preferably using a timeline diagram or flow chart)<br>c) Documents degree of success in implementing intervention components)<br>d) Describes how and why the initial plan evolved, and the most important lessons learnt from that evolution, particularly the effects of internal feedback from tests of change (reflexiveness)<br><br>Changes in processes of care and patient outcomes associated with the intervention: 1) Presents data on changes observed in the care delivery process 2)Presents data on | a) Basic characteristics listed in table 2<br>b) flow diagram showed in figure 1.<br>c) indicate the complete rate of above 80% (Line 210-213)<br>d) address in the introduction and reference the paper that been published in the same institution (Ref.13)<br><br><br>1)presents the different variables required to derive the new tool (see results Line 229-235)<br>2)see detail in outcome measurement (see appendix table 1,2,3, Appendix figure 1,2,3)<br>3) N/A<br>4)see detail in outcome measurement (Line 246-278)<br>5) missing data discussed in limitation section (Line 392-397) | Yes |

| | | | |
|---|---|---|---|
| | changes observed in measures of patient outcome (eg, morbidity, mortality, function, patient/staff satisfaction, service utilisation, cost, care disparities) 3) Considers benefits, harms, unexpected results, problems, failures) 4)Presents evidence regarding the strength of association between observed changes or improvements and intervention components or context factors) 5) Includes summary of missing data for intervention and outcomes | | |
| 14.Summary | *a)* Summarises the most important successes and difficulties in implementing intervention components, and main changes observed in care delivery and clinical outcomes<br>*b)* Highlights the study's particular strengths | a) report and summarized in Line 301-310<br>b) report the study's particular strengths Line 307-310 | Yes |
| 15. Relation to other evidence | Compares and contrasts study results with relevant findings of others, drawing on broad review of the literature; use of a summary table may be helpful in building on existing evidence | This was discussed broadly in the discussion section (Line 311-371) | Yes |
| 16.Limitations | *a)* Considers possible sources of confounding, bias, or imprecision in design, measurement, and analysis that might have affected study outcomes (internal validity) *(b)* Explores factors that could affect generalisability (external validity)—eg, representativeness of participants, effectiveness of implementation, dose-response effects, features of local care setting*(c)* Considers likelihood that observed gains may weaken over time and describes plans, if any, for monitoring and maintaining improvement; explicitly states if such planning was not done*(d)* Reviews efforts made to minimise and adjust for study limitations*(e)* Assesses the effect of study limitations on interpretation and application of results | a) address the population selection bias (Line 376-378)<br>b) consider the future large multi-center study (Line 382-383)<br>c) report the limitation of sample size and missing data (Line 390-395)<br>d) report the limitation of outcome measurements(Line 389-390)<br>e) report the limitation of ED setting of using SONET tools and its interpretation (Line 397-399) | Yes |
| 17.Interpretation | *a)* Explores possible reasons for differences between observed and expected outcomes*(b)* Draws inferences consistent with the strength of the data about causal | a) reported and discussed on variables selection and discussed broadly when compared with different previous studies (Line 311-331) | Yes |

| | | | |
|---|---|---|---|
| | mechanisms and size of observed changes, paying particular attention to components of the intervention and context factors that helped determine the intervention's effectiveness (or lack thereof), and types of settings in which this intervention is most likely to be effective*(c)* Suggests steps that might be modified to improve future performance *d)* Reviews issues of opportunity cost and actual financial cost of the intervention | b) discussed the confounders and collinear factors (Lien 311-331)<br>c) suggesting the traffic light reporting system to simplify the ED overcrowding reporting system (Line 332-344)<br>d) N/A | |
| 18.Conclusions | *a)* Considers overall practical usefulness of the intervention<br>*b)* Suggests implications of this report for further studies of improvement interventions | a) reported in the conclusion (Line 403-404)<br>b) indicate the simplified traffic light system (Line 404-406) | Yes |
| 19. Funding | Describes funding sources, if any, and role of funding organisation in design, implementation, interpretation, and publication of study | N/A | N/A |
